# Case report: Metastatic metaplastic breast cancer with choriocarinomatous features: Targeting the choriocarcinoma component for cure

**DOI:** 10.3389/fonc.2022.1061679

**Published:** 2023-01-05

**Authors:** Krisha K. Mehta, Rosa Nouvini, Jingxuan Liu, Yi Wang, Alison Stopeck

**Affiliations:** ^1^ Department of Medicine, Stony Brook University, Stony Brook, NY, United States; ^2^ Department of Medicine, Memorial Sloan Kettering Cancer Center, New York, NY, United States; ^3^ Department of Pathology, Stony Brook University, Stony Brook, NY, United States; ^4^ Department of Medicine, Stony Brook Cancer Center, Stony Brook University, Stony Brook, NY, United States

**Keywords:** breast cancer with choriocarcinomatous features, choriocarcinoma, metaplastic breast cancer, chemotherapy, methrotrexate, long term remission

## Abstract

Breast cancer with choriocarcinomatous features (BCCF) is a rare and aggressive breast cancer. BCCF carries a poor prognosis and there is unfortunately scant literature to guide treatment beyond surgical resection with most patients receiving standard regimens for breast cancer. In our case, we present a 42-year-old female with an initial hCG of 2,324 and two suspicious lesions of the right breast. On biopsy, each lesion had distinct histopathology with the larger lesion diagnosed as BCCF and the smaller lesion being an invasive ER/PR positive ductal carcinoma. The diagnosis of BCCF rather than metastatic choriocarcinoma was confirmed using DNA typing. Salvage chemotherapy targeting choriocarcinoma resulted in marked clinical and biomarker success including normalization of the hCG. After recurrent brain metastases were diagnosed, high dose chemotherapy with methotrexate was administered resulting in long term remission.

## Background

Breast cancer with choriocarcinomatous features (BCCF) can be challenging to treat. In all cases, the diagnosis of choriocarcinoma should be excluded in the reproductive organs prior to attributing the breast findings to a metaplastic breast cancer. BCCF was first described in 1981 by Saigo and Rosen and since then there have been at least 18 cases reported in the literature (1). Histology and immunohistochemistry reveal human chorionic gonadotropin (hCG) positive choriocarcinomatous cells (syncytiotrophoblasts and cytotrophoblasts) mostly in the background of DCIS or invasive carcinoma (2). The choriocarcinomatous area is usually surrounded by hemorrhage and necrosis. Serum hCG is variably expressed in BCCF. When hCG is elevated, it serves as an accurate marker for disease recurrence and response to treatment (3). BCCF carries a poor prognosis and there is unfortunately scant literature to guide treatment beyond surgical resection with most patients receiving standard regimens for breast cancer. In our case, we present a 42-year old female diagnosed with BCCF and recurrent brain metastases who is now disease free over 5 years from her last treatment after progressing through standard breast cancer regimens and responding to chemotherapy appropriate for choriocarcinoma.

## Case presentation

A 42-year-old Gravida 3 Para 2 female presented post-op day #4 from a dilation and curettage (D+C) for a presumed missed abortion based on elevated hCG. Subsequent hCG increased from 2,324mIU/mL to 2,445 after D+C and she was sent to the hospital for evaluation of a suspected ectopic pregnancy. Endovaginal ultrasound revealed a complex subcentimeter cystic structure adjacent to the right ovary suspicious for an ectopic pregnancy. She received one dose of methotrexate (MTX) 0.5mg/kg with a second dose of MTX administered day 4 secondary to a rising hCG. Her hCG continued to rise and she underwent laparascopic surgery which revealed an edematous right fallopian tube which was excised, revealing benign pathology.

**Figure 1:**
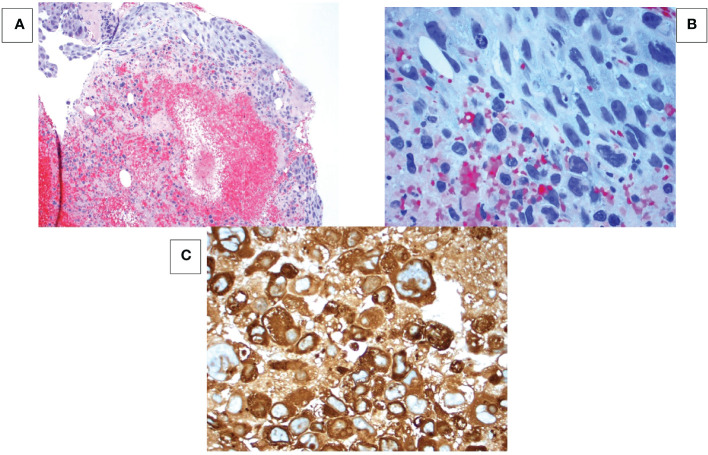
Breast mass at 10 o’clock position. **(A)** Hematoxylin-eosin (HE) stain at 100x magnification shows tumor cells in a hemorrhagic necrotic background. **(B)** HE stain at 400x magnification shows sheet-like formation of epithelial cells with pleomorphic hyperchromatic nuclei, multiple prominent nucleoli and abundant vacuolated cytoplasm. No syncytiotrophoblastic cells are seen. **(C)** Immunohistochemistry shows all cancer cells strongly express human chorionic gonadotropin (hCG).

**Figure 2:**
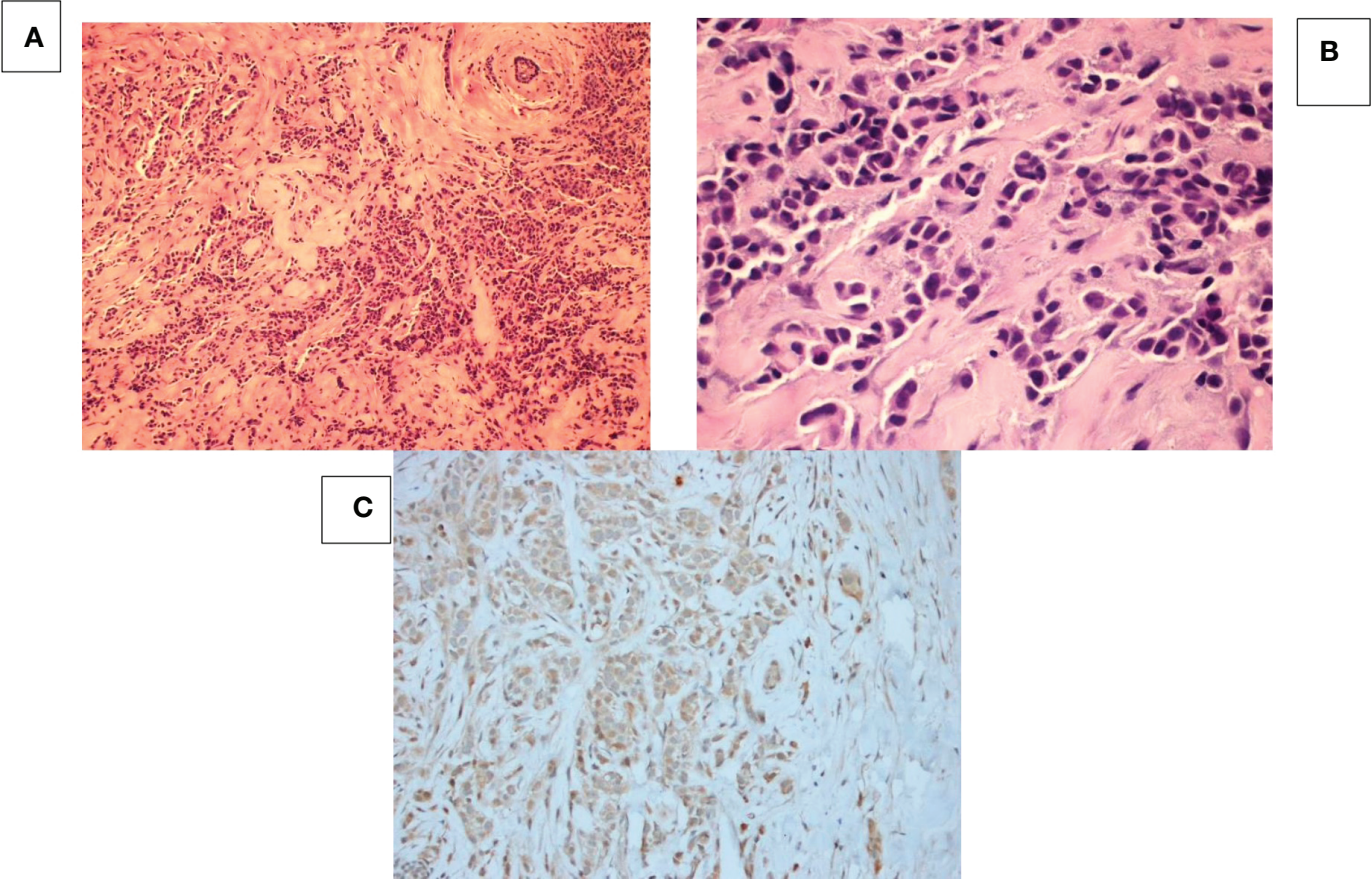
Breast mass at 6 o’clock position. **(A)** HE staining at 100x magnification shows tumor cells with sheets, tubular, cords or single cell formation infiltrating through desmoplastic stroma. **(B)** HE staining at 400x magnification shows moderately differentiated tumor cells with high nuclear: cytoplasmic ratio and pleomorphic hyperchromatic nuclei infiltrating through desmoplastic stroma. **(C)** hCG IHC shows that all the cancer cells are negative for beta-HCG.

In the interim, the patient self-palpated a right breast mass and underwent mammography which revealed a high density mass in the right axillary tail. Bilateral breast magnetic resonance imaging (MRI) revealed an approximately 6.0cm mass in the 10 o’clock position abutting the pectoral muscle with two additional lesions measuring approximately 6mm that were 3mm and 1.2mm inferior to the main tumor mass highly suggestive of satellite lesions. Also found was a 6mm irregularly shaped enhancing lesion at 6 o’clock and an 8mm rounded right axillary lymph node suspicious for metastases. Core needle biopsy of the 6mm lesion at 6 o’clock revealed invasive ductal carcinoma, moderately differentiated, ER 80%, PR 80%, Ki-67 15%, and Her2 negative. GATA 3, cytokeratin 7, and mammoglobin were expressed, and the tumor cells did not express hCG as demonstrated in **Figure 1**. Core biopsy of the dominant 6cm mass at 10 o’clock revealed a very different picture with a poorly differentiated carcinoma with extensive hemorrhage and necrosis observed. Immunohistochemical stains found the tumor cells did not express estrogen, progesterone, or HER2 receptors (triple negative) with a markedly elevated proliferative marker, Ki-67, at 70%, with expression of cytokeratin 7, GATA3, and importantly, hCG as demonstrated in **Figure 2**. She underwent staging with ^18^F-FDG PET/CT (positron emission tomography with computed tomography) which revealed a large hypermetabolic (SUV 7.3) right breast mass measuring 4.5 x 3.0 cm with central necrosis, inflammatory skin changes, and several small mildly hypermetabolic right axillary lymph nodes concerning for early metastatic disease. No other sites of disease or abnormal uptake were noted. A brain MRI revealed no evidence of metastatic disease.

She initiated neoadjuvant treatment with dose dense AC [Adriamycin (60mg/m2) and Cyclophosphamide (600mg/m2)]. Clinically, there was no response to therapy and her hCG increased to 12,580mIU/mL after 2 cycles on treatment. Her therapy was then switched to a known salvage regimen for choriocarcinoma and she received alternating cycles of paclitaxel 135mg/m2 (day 1) and cisplatin 60mg/m2 (day 1) with paclitaxel 135mg/m2 (day 15) and etoposide 150mg/m2 (day 15) (TP/TE), for a total of 8 cycles (4 cycles of each treatment). Her hCG decreased markedly after her first cycle, as did her breast mass by physical exam. After completing 4 months of therapy, her hCG had normalized and her breast MRI revealed a partial response to treatment with no significant change in the smaller ER+ breast cancer at 6:00. She underwent a right modified radical mastectomy with a left risk reducing (prophylactic) mastectomy performed per patient wishes. Pathology of the BCCF cancer at 10:00 showed a 4.7cm necrotic mass without viable tumor cells. At the 6:00 position, two adjacent foci of invasive ductal carcinoma were seen, measuring 1.5cm and 2.3mm. The larger lesion was moderately differentiated, ER 29%, PR negative, HER2 negative, with a low Ki-67 at 3% and neither area expressed hCG by immunohistochemical analysis. Four of thirteen axillary lymph nodes contained metastatic disease with the largest metastatic focus at 2.5mm. Biomarkers on the involved lymph node revealed a high grade triple negative cancer with an elevated Ki-67 of 61%. Of note, hCG was not expressed in the lymph node by immunohistochemistry. DNA typing of the tumor was performed to confirm a primary breast cancer rather than metastatic choriocarcinoma. The tumor revealed only maternal DNA with no paternal DNA present confirming metaplastic breast cancer. Genetic testing of the patient did not reveal a deleterious BRCA mutation.

One week following her breast surgery and 3 weeks after completing neoadjuvant chemotherapy, she presented with a grand mal seizure and was found to have a hemorrhagic lesion measuring 2 cm in the left temporoparietal lobe associated with vasogenic edema. Her hCG level had increased from 2 to 48mIU/mL. She underwent left temporal craniotomy with tumor excision. Pathology revealed a high-grade metastatic carcinoma with hCG expression. She received a single stereotactic radiosurgery fraction of 15 Gy to the brain and comprehensive radiation treatment to the right chest wall and regional nodal areas (50 Gy in 25 fractions to the right chest wall, supraclavicular, axilla, and internal mammary nodes).

Two months after neurosurgery and stereotactic radiation, she was re-admitted to the hospital with recurrent neurologic findings. Her hCG had increased from 1 to 139mIU/mL. Repeat MRI showed hemorrhage in the surgical bed. She underwent re-resection of the brain cavity and a small foci of residual/recurrent hCG expressing carcinoma was seen on pathology. She then went on to receive a total of five cycles of high dose MTX (3.5gm/m2 per cycle). Her hCG became undetectable after 3 cycles. Restaging PET/MRI prior to her final cycle of high dose methotrexate did not show any evidence of disease outside the brain. She was started on anastrazole one month after completion of high dose MTX. She is now over 5 years from completion of chemotherapy and 5 years from diagnosis and her hCG remains undetectable, her brain MRI shows only stable post-operative findings, and she is without evidence of active disease.

## Discussion

BCCF, a rare, aggressive and unique entity, is a cancer characterized by cells including multinucleated synctiotrophoblast-like giant cells expressing hCG and one whose pathogenesis and optimal treatment remains undefined. Metaplastic changes associated with breast cancer include osseous, chondroid, matrix production, squamous, and spindle cells (4). It is thought that through a metaplastic process in carcinomatous breast cells, commonly infiltrating ductal carcinoma cells, choriocarcinomatous cells are formed. Metaplasia to choriocarcinoma has been seen in other adenocarcinomas as well including cancers of the esophagus, stomach and colon (5).

On gross examination, the tumor generally appears well circumscribed with hemorrhagic and necrotic components, similar to choriocarcinoma with breast metastasis. Typically, the choriocarcinomatous cells are associated with hemorrhage and necrosis in a background of either invasive ductal carcinoma or ductal carcinoma *in situ* (6). Choriocarcinomatous cells have been described as multinucleated, giant bizarre looking cells resembling syncytiotrophoblastic and cytotrophoblastic cells (7). Interestingly our case did not have the typical histologic pattern of BCCF as there was no component of invasive or *in situ* ductal carcinoma or multinucleated giant cells resembling syncytiotrophoblastic cells. However, some of the mononuclear tumor cells did resemble cytotrophoblastic cells. In addition, there was a second focus of typical invasive ductal breast cancer without hCG expression located in a different quadrant of the breast which may have been a second primary lesion versus potentially the precursor lesion for the BCCF. On immunohistochemistry, the choriocarcinomatous cells stain positive for hCG as in our case. It is important to note that 5 -21% of ductal carcinoma cells can also express hCG positivity on immunohistochemistry (4); and hCG expression alone does not confirm a BCCF diagnosis. Serum hCG is commonly elevated in BCCF. It can also be elevated in up to 12 – 33% of breast cancer patients who do not have evidence of choriocarcinoma or choriocarcinoma features in their tumor (1). ^18^F-FDG PET/CT has also been used as a tool for detecting malignant tissue due to malignant cells accumulating FDG as a result of their high rates of glycolysis (8). Sung et al. used ^18^F-FDG PET/CT to diagnose the tumor mass in one of their BCCF cases (8). Similarly, in our case ^18^F-FDG PET/CT revealed a large hypermetabolic right breast mass measuring 4.5 x 3.0 cm and hypermetabolic right axillary lymph nodes consistent with her disease presentation.

BCCF must also be differentiated from metastatic choriocarcinoma to the breast. Patients with metastatic choriocarcinoma typically have a history of reproductive tumors and a primary breast tumor is not present (6). While uncommon, trophoblastic cancers can metastasize to the breast. DNA typing can be useful to exclude metastatic gestational trophoblastic disease and help differentiate choriocarcinoma versus BCCF. Choriocarcinoma, as a gestational cancer arising from hydatiform moles, carries both paternal and maternal genetic DNA. Thus, DNA genotyping is a definitive tool for distinguishing metastatic gestational trophoblastic cancer from other somatic cancers that mimic gestational tumors (9). As our patient’s tumor consisted of only maternal DNA, we excluded a gestational origin to the cancer. To our knowledge, this is one of the first case reports that used DNA typing to differentiate between metastatic choriocarcinoma and BCCF.

As in most metaplastic breast cancers, immunohistochemistry is typically triple negative (minimal to no ER, PR, or HER2/neu expression) in BCCF. Siddiqui et al. did present a case in which there was immunohistochemical evidence of expression of both ER and PR in the background tumor with PR positivity and ER negativity in the giant cells. The lack of hormone receptor positivity adds to the complexity in treatment and disease-free survival. To add to the poor prognosis, patients with BCCF also present at a younger age (average age 48 years) and most present with palpable masses in the right breast (10). One theory to explain the aggressive clinical course characteristic of BCCF is that pregnancy associated proteins such as hCG suppress the immune system to protect materno-fetal immune reactivity and thereby permit cancer cells to evade host immunosurveillance (10).

The development of hemorrhagic brain metastases is another hallmark of choriocarcinoma, and our patient developed a symptomatic, hemorrhagic brain metastasis soon after stopping neoadjuvant chemotherapy. It is likely this metastasis was present at diagnosis, despite the normal brain MRI on staging, and suggests patients with BCCF often have a clinical course similar to patients with gestational trophoblastic.

Treatment of BCCF has not been well established. Unlike choriocarcinoma which has a favorable prognosis in the non-metastatic setting and responds well to chemotherapy, BCCF has a poor prognosis with variable survival rates depending on stage. Endocrine therapy, chemotherapy and surgery are the commonly used treatment strategies for BCCF currently. Thus far, endocrine therapy such as tamoxifen or gonadotropin-releasing hormone analogues, has not shown significant effectiveness in the treatment of BCCF (10). Surgery has generally been proven to be effective in treating BCCF’; though there are many cases in which patient develop multiple metastases shortly after surgery (11). There are also other cases reported in which BCCF patients have a disease-free survival of 1 year or more after undergoing surgical resection (2, 12). Chemotherapy is often used as an adjuvant therapy to surgical resection with variable success. Standard breast cancer regimens seem to be relatively ineffective and regimens targeting the choriocarcinoma component (1), as in our case, may be more effective (see **Table 1**). Interestingly, capecitabine has also been described in at least one case report as an effective regimen in a case of refractory BCCF (10); and while not a standard regimen for choriocarcinoma it has been used as a salvage regimen in gestational trophoblastic neoplasia previously with success. In our case, we used salvage chemotherapy against choriocarcinoma (paclitaxel, cisplatin and etoposide) with marked clinical and biomarker success including normalization of the hCG after four months of treatment. After recurrent brain metastases were diagnosed, high dose chemotherapy with methotrexate was used as treatment and she currently has no evidence of disease over 5 years after receiving her final cycle of the chemotherapy. This is the first case to show the potential effectiveness of methotrexate in treating metastases to the brain from breast cancer with choriocarcinomatous features.

**Table 1:** Previous case reports of BCCF with details of case, treatment and outcome.

Study (year)	Age (sex)	Pregnant (y/n)	Histology	Initial Serum bHCG	Stage	Treatment	PFS	OS
Saigo PE, 1981	55 (F)	N	IDC*, with co-existing anaplastic areas associated with necrosis & hemorrhage, choriocarcinomatous cells found in hemorrhagic area	N/A*****	IIA (T2N0M0)	Left radical mastectomy	N/A	7 months
Fowler CA, 1995	32 (F)	Y (mass felt during 3^rd^ trimester & presented 6wks post partum)	Large mass w/ necrotic center, choriocarcinoma	9,920	IV (lung)	10 cycles Etoposide, MTX, Adriamycin, Vincristine, Cyclophosphamide	9 months	1 year
Murata T, 1999	38 (F)	N	Large-sized, oval-shaped tumor cells with occasional hemorrhagic necrosis. MGCs*** resembling syncytiotrophoblasts. DCIS**** background,	N/A	IIIA (T3N2M0)	Right MRM f/b radiation therapy and adjuvant 5FU derivative & anti-GnRH	2 months	7 months
Resetkova E, 2004	38 (F)	Y	Syncytial pattern of cells surrounded by rim of chronic lymphoplasmacytic infiltrate. Multiple atypical mitotic figures & focal tumor necrosis	<1	IB (T1bN0M0)	Excisional bx with clear margins, elective abortion, chemo (unknown)	1 year	N/A
Resetkova E, 2004	54 (F)	N	IDC with features of metaplastic ca. with a component of MGCs with a syncytiotrophoblast-like appearance. Adjacent necrosis & hemorrhage.	3.4	IIB (T3N0M0)	MRM, 2 cycles adjuvant chemo (unknown) + XRT R chest wall	6 months	N/A
Siddiqui NH, 2006	56 (F)	N	IDC & loosely cohesive giant cells with areas of hemorrhage. ER+/PR+ in background tumor cells and ER-/PR+ in the giant tumor	N/A	IIA (T2N0M0)	Right partial mastectomy f/b left breast radical mastectomy		7 months
Akbulut M, 2008	53 (F)	N	IDC with areas of necrosis & multinucleated syncytiotrophoblastic type giant cells and cytotrophoblast looking cells	N/A	Stage IIA (T2N0M0)	Left partial mastectomy with ALND		6 years
Akbulut M, 2008	50 (F)	N	IDC with numerous MGCs, tumor cells resembling syncytiotrophoblastic and cytotrophoblastic cells, extensive necrosis and hemorrhage	N/A	Stage IIA (T2N0M0)	Right radical mastectomy		4 years
Zhu Y, 2014	32 (F)	N	MGCs resembling syncytiotrophoblasts. Extensive hemorrhage, no infiltrating ductal ca or DCIS.	22,931	IV (lung, kidney)	**2 cy docetaxel + epirubicin →POD → 2cy docetaxel + cis → 1 cy docetaxel + Lobaplatin →toxicity → 3cy docetaxel + capecitabine → 9 cy capeceitabine	37 months	N/A
Oguz A, 2014	69 (F)	N	IDC with most of tumor with choriocarcinomatous differentiation with perineural invasion	N/A	IA (T1N0M0)	Left MRM f/b 6 cy cyclophosphamide (500mg), doxorubicin (50mg), 5FU (500mg/m2)	23 months	N/A

*IDC, invasive ductal carcinoma; MTX, methotrexate; MRM, modified radical mastectomy; Cis, cisplatin,

** Doses of chemo used in this study were as follows: Q21day cycles, Docetaxel 75mg/m2 (1x/cycle), Epirubicin 75mg/m2 (1x/cycle), Cisplatin 75mg/m2 (1x/cycle), Lobaplatin 35mg/m2 (1x/cycle), Capecitabine 2g/m2 (2wk on, 1wk off)

***MGC, multinucleated giant cell

****DCIS, ductal carcinoma in situ

*****N/A – Not reported

In summary, hCG when elevated, is a reliable tumor marker for disease monitoring in BCCF patients and DNA typing is a reliable confirmatory test for the diagnosis of BCCF versus metastatic choriocarcinoma. The successful treatment of our patient suggests therapies that target the metaplastic component of breast cancer, choriocarcinoma in our case, can be more effective than standard breast cancer regimens and should be considered early in the course of therapy in nonresponsive patients.

## Data availability statement

The datasets presented in this article are not readily available because All PHI has been excluded in the article. Requests to access the datasets should be directed to KM, krisha.mehta@stonybrookmedicine.edu.

## Author contributions

All authors listed have made a substantial, direct, and intellectual contribution to the work and approved it for publication.
